# Congenital Pyloric Atresia with Distal Duodenal Atresia- Role of CT Scan

**Published:** 2014-07-10

**Authors:** Yogender Singh Kadian, KN Rattan

**Affiliations:** Department of Paediatric Surgery, PGIMS, Rohtak

**Keywords:** Pyloric atresia, CT scan

## Abstract

The mainstay of diagnosis of congenital pyloric atresia is by plain X-ray of the abdomen showing a large gas bubble with no gas distally. But very rarely it can be associated with distal duodenal atresia when the baby may present as lump abdomen. In such a situation apart from the X-ray, another radiological investigation is needed to delineate the exact nature of the lump. Since the role of ultrasonography is limited in intestinal pathologies and contrast studies are not informative in atresias, the CT scan is the ideal choice. We had managed a case of pyloric atresia with similar presentation with preoperative CT scan.

## INTRODUCTION

Congenital pyloric atresia (CPA) is a rare condition which can occur in isolation or have associated anomalies (1,2). The mainstay of its diagnosis is by plain X-ray of the abdomen showing a large gas bubble with no gas distally. However, when it is associated with distal duodenal atresia, the baby may present with a lump abdomen (3). We are reporting a similar case of pyloric atresia with distal duodenal atresia, but we did a preoperative CT scan, which showed a hugely dilated duodenum. This case is reported to highlight the role of CT scan in pyloric atresia and probably the first case of pyloric atresia-distal duodenal atresia with preoperative CT picture. 

## CASE REPORT

A 2.2 Kg full term male newborn born to a 21-year-old primigravida mother through normal vaginal delivery and was referred with complaints of non-bilious vomiting and abdominal distenson. Examination revealed a lump about 11x9 cm size in the right side of abdomen. The plain radio-graph of the abdomen showed only the gastric bubble, with the rest of the abdomen being opaque (Fig.1). The patient was started intravenous fluids and antibiotics. A CT scan of the abdomen was also done which revealed a dilated fluid filled gut loop and rest of the organs were normal (Fig. 2). At surgery, the patient had pyloric atresia, duodenal atresia at duodeno-jejunal flexure along with massive dilation of the duodenum, and atresia in the ileum. Mickulicz procedure for the pyloric atresia, and reduction duodenoplasty along with duodenojejunostomy for the duodenal atresia were performed. The portion of ileum with atretic segment was resected and ileostomy was done. A penrose drain was put after a thorough peritoneal lavage. The baby was administered broad-spectrum antibiotics including cefotaxime, meropenum and metronidazole and parentral nutrition. The baby started moving ileostomy on 5th postoperative day, but the general condition did not improve and he developed sclerema and other features of sepsis. The parents took the baby to another center against medical advice and never returned in follow up, it is presumed that baby might have died.

**Figure:**
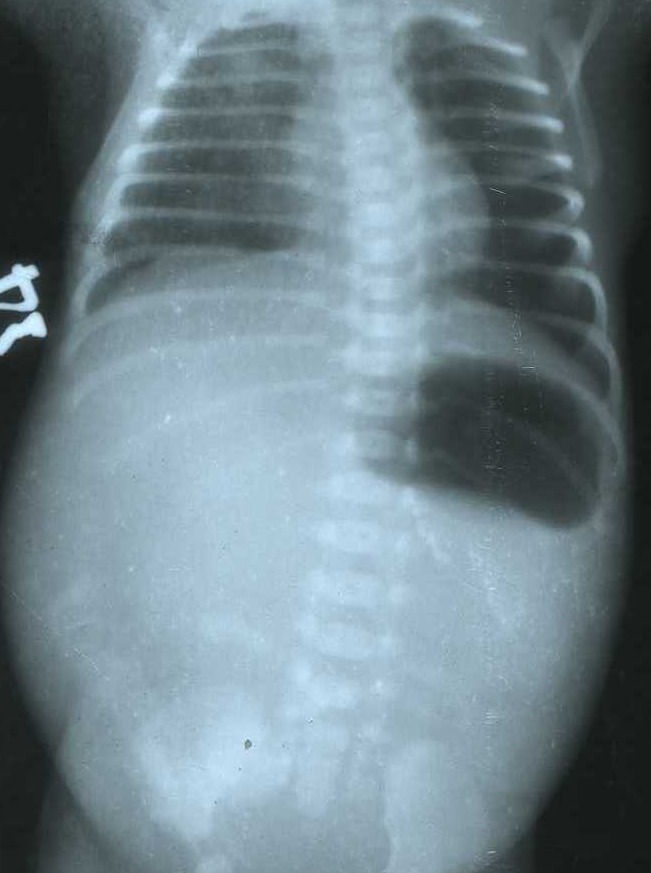
Figure 1: A plain X-ray of the abdomen showing a single large gastric air bubble with no gas distally.

**Figure:**
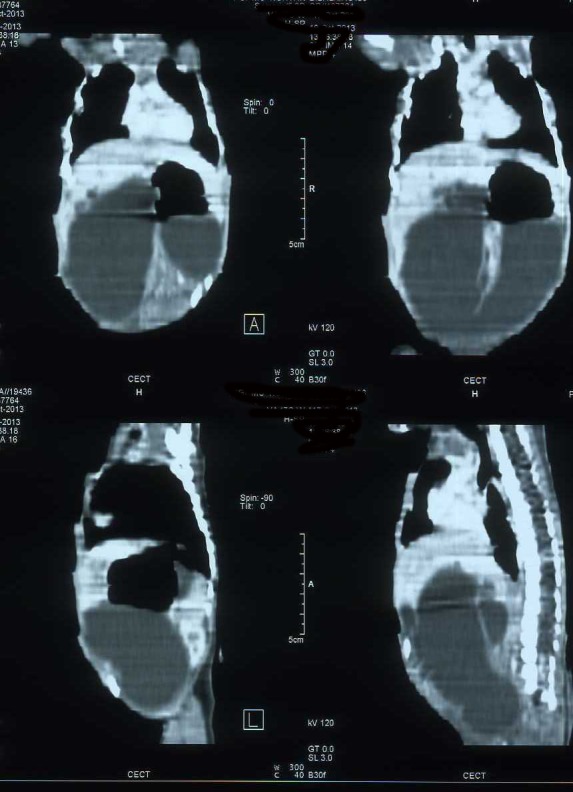
Figure 2: CT scan of the abdomen showing a dilated fluid filled gut loop.

## DISCUSSION

Congenital pyloric atresia is thought to result from development arrest between the 5th and 12th week of intrauterine life (4). It can occur in isolation or can have associated anomalies in the range of 40-50% of cases which can be epidermolysis bullosa (EB), aplasia cutis congenital, duodenal atresia, ileal/colonic atresia and duplication cysts (2,5). Anatomically, it is divided into three types Type1 is pyloric web or membrane or web , type 2 is pyloric atresia with a solid cord between the two ends and type 3 is pyloric atresia with a gap between two the stomach and duodenum (2). The overall survival of this condition has improved dramatically in isolated cases but same is not true when it is associated with other anomalies (5,6). 

The diagnosis of pyloric atresia is smple and based on the presence of a single large gastric air bubble with no gas distally. However in some patients there is association of distal duodenal atresia along with pyloric atresia, this can lead to a closed duodenal loop with accumulation of biliary and pancreatic secretions leading to massive dilatation of the duodenum (3,4). This subset of patients can present as perforation peritonitis (rupture of overdistended duodenum) or lump abdomen as in the present case. In such a situation, another investigation is needed to adequately delineate the anatomy of the lesion for proper management of the patient. Since the role of ultrasonography of abdomen is limited in intestinal pathologies and contrast studies has not been found to be informative in cases of intestinal atresias (2,5), the CT scan is the ideal choice of radiological investigation (7). The abdominal CT scan can also differentiate between the dilated duodenal loop or any associated duplication cyst or any other associated lesion.

The principle of surgical treatment of pyloric atresia is restoring the continuity of the stomach and duodenum by various well described procedures (2,5,6). However in case of associated distal duodenal atresia, the tapering duodenoplasty and duodenojejunostomy is also needed (3) .

The overall survival of isolated pyloric atresia is good but when associated with multiple atresias, survival has not been reported till date. The present case also could not survive.

The purpose of presenting this manuscript is to illustrate the role of CT scan in pyloric atresia especially when associated with a distal duodenal atresia and presenting as a lump.

## Footnotes

**Source of Support:** Nil

**Conflict of Interest:** None

